# Changes in primary metabolism under light and dark conditions in response to overproduction of a response regulator RpaA in the unicellular cyanobacterium *Synechocystis* sp. PCC 6803

**DOI:** 10.3389/fmicb.2015.00888

**Published:** 2015-08-26

**Authors:** Hiroko Iijima, Tomokazu Shirai, Mami Okamoto, Akihiko Kondo, Masami Yokota Hirai, Takashi Osanai

**Affiliations:** ^1^School of Agriculture, Meiji University, KawasakiJapan; ^2^RIKEN, Center for Sustainable Resource Science, YokohamaJapan; ^3^Department of Chemical Science and Engineering, Graduate School of Engineering, Kobe University, KobeJapan

**Keywords:** amino acids, cyanobacteria, *Synechocystis*, response regulator, sugar metabolism

## Abstract

The study of the primary metabolism of cyanobacteria in response to light conditions is important for environmental biology because cyanobacteria are widely distributed among various ecological niches. Cyanobacteria uniquely possess circadian rhythms, with central oscillators consisting from three proteins, KaiA, KaiB, and KaiC. The two-component histidine kinase SasA/Hik8 and response regulator RpaA transduce the circadian signal from KaiABC to control gene expression. Here, we generated a strain overexpressing *rpaA* in a unicellular cyanobacterium *Synechocystis* sp. PCC 6803. The *rpaA*-overexpressing strain showed pleiotropic phenotypes, including slower growth, aberrant degradation of an RNA polymerase sigma factor SigE after the light-to-dark transition, and higher accumulation of sugar catabolic enzyme transcripts under dark conditions. Metabolome analysis revealed delayed glycogen degradation, decreased sugar phosphates and organic acids in the tricarboxylic acid cycle, and increased amino acids under dark conditions. The current results demonstrate that in this cyanobacterium, RpaA is a regulator of primary metabolism and involved in adaptation to changes in light conditions.

## Introduction

Cyanobacteria are organisms performing oxygenic photosynthesis that exist in various environmental niches such as fresh water, seawater, soil, and hot springs. Studying the regulatory mechanism of cyanobacterial metabolism is important in environmental biology and biotechnology. One of the most widely studied cyanobacteria is the non-nitrogen fixing species *Synechocystis* sp. PCC 6803 (hereafter *Synechocystis* 6803). *Synechocystis* 6803 cells grow fast and are naturally transformable with homologous recombination (Berla et al., 2013).

Cyanobacteria have a circadian rhythm and their central oscillator consists of three proteins KaiABC, first found in the unicellular cyanobacterium *Synechococcus* sp. PCC 7942 (hereafter *Synechococcus* 7942; Ishiura et al., 1998). An important issue for study in the circadian system is metabolic compensation, which is the persistence and entrainment of circadian rhythms in response to various nutrient conditions (Johnson and Egli, 2014). KaiC is an enzyme that phosphorylates and dephosphorylates its own residues in a strict order (Nishiwaki et al., 2007; Rust et al., 2007). ATP is a substrate of KaiC phosphorylation, and the ATP/ADP ratio is important for entrainment of the circadian clocks (Rust et al., 2011). KaiC phosphorylation is inhibited by ADP and integrates metabolic information into KaiC through the kinase-stimulation activity of KaiA (Rust et al., 2011). ATP is decreased under dark conditions and treatment with a dark pulse leads to a phase shift in the circadian clock (Rust et al., 2011). Oxidized quinones also input signals into the circadian clock in *Synechococcus* 7942 via the inhibition of KaiC phosphorylation (Kim et al., 2012). Thus, information on the availability of biochemical energy and light/dark conditions is transduced into circadian clocks. Glycogen metabolism entrains the circadian oscillator by providing ATP, and the mutants deficient in glycogen synthesis show the phenotype of a circadian clock that is hypersensitive to dark pulses (Pattanayak et al., 2014).

SasA is a histidine kinase interacting with KaiC in *Synechococcus* 7942 (Iwasaki et al., 2000). SasA is autophosphorylated by its histidine residue and the phosphate moiety is transferred to the cognate response regulator RpaA (Takai et al., 2006). The gene *rpaA* is the “regulator of phycobilisome association,” whose protein is involved in energy transfer from phycobilisome to Photosystem I (Ashby and Mullineaux, 1999). The phosphorelay from SasA to RpaA is enhanced in the presence of KaiC (Takai et al., 2006). The knockout mutants of *sasA* or *rpaA* in *Synechococcus* 7942 grow normally under continuous light conditions, but the growth is severely retarded under light/dark cycle conditions (Takai et al., 2006). The knockout of *rpaA* alters the gene expression in *Synechococcus* 7942 widely; the genes, whose peaks of the expression are subjective dusk or dawn, are down-regulated or up-regulated, respectively, in the *rpaA* mutant (Markson et al., 2013). Chromatin-immunoprecipitation with high throughput sequencing (ChIP-Seq) analysis reveals 110 binding sites in the *Synechococcus* 7942 genome, which has A/T-rich motif included in the promoters of *kaiBC* and *rpoD6* (encoding an RNA polymerase sigma factor). *In vitro* assay demonstrates that phosphorylated RpaA binds with the promoter regions, while non-phosphorylated RpaA does not (Hanaoka et al., 2012; Markson et al., 2013).

The mechanistic implications of a circadian clock in *Synechocystis* 6803 have remained obscure due to the redundancy of *kaiABC* genes, with one *kaiA*, three *kaiB* (*kaiB1*–*B3*), and three *kaiC* (*kaiC1*–*3*; Kanesaki et al., 2012). The *kaiAB1C1* and *kaiC2B2* genes constitute an operon in the *Synechocystis* 6803 genome. KaiC1 phosphorylation is dependent on KaiA and KaiC directly interacting with KaiA (Wiegard et al., 2013). The phosphorylation of KaiC2 and KaiC3 is not dependent on KaiA, and therefore KaiAB1C1 proteins seem to be the central oscillator in *Synechocystis* 6803 (Wiegard et al., 2013). *Synechocystis* 6803 contains SasA (Hik8, sll0750) and RpaA (Rre31, slr0115) orthologs. Hik8 interacts with KaiC1, but not KaiC2 *in vivo* (Osanai et al., 2015). The knockout of *hik8* results in pleiotropic phenotypes, with the gene expression of enzymes in the glycogen catabolism, glycolysis, and the oxidative pentose phosphate (OPP) pathway altered (Singh and Sherman, 2005). The *hik8* overexpression also leads to changes in primary metabolism (Osanai et al., 2015). Glycogen and sugar phosphate levels are decreased under light conditions and amino acid levels such as glycine and lysine are increased by *hik8* overexpression (Osanai et al., 2015). The involvement of RpaA in salt and hyperosmotic stress has been shown by microarray, with the knockout of *rpaA*/*rre31* down-regulating the salt-induced gene expression (Shoumskaya et al., 2005). Nevertheless, in these studies, the involvement of RpaA in the regulation of primary metabolism and the effect of *rpaA* modification on metabolic alteration has remained unclear due to lack of metabolome data.

Here, we generated a *Synechocystis* 6803 strain overexpressing *rpaA*. The *rpaA*-overexpressing strain showed pleiotropic and similar phenotypes with the *hik8*-overexpressing strain. Genetic and metabolomic analyses indicate that RpaA plays pivotal roles in metabolic regulation under both light and dark conditions.

## Materials and Methods

### Bacterial Strains and Culture Conditions

The glucose-tolerant (GT) strain of *Synechocystis* sp. PCC 6803, isolated by Williams (1988), and the *rpaA*-overexpressing strain, designated as ROX310, were grown in modified BG-11 medium, which consisted of BG-11_0_ liquid medium (Rippka, 1988) containing 5 mM NH_4_Cl (buffered with 20 mM HEPES-KOH, pH 7.8). Among GT substrains, the GT-I strain was used in this study (Kanesaki et al., 2012). Liquid cultures were bubbled with 1% (v/v) CO_2_ in air and incubated at 30°C under continuous white light (ca. 50–70 μmol photons m^-2^ s^-1^). Growth and cell densities were measured at OD_730_ with a Hitachi U-3310 spectrophotometer (Hitachi High-Tech., Tokyo, Japan). Kanamycin (10 μg/mL) was added to ROX310 during pre-culture.

### Construction of Plasmids for *rpaA* Overexpression

A region of the *Synechocystis* 6803 genome encoding the *rpaA* (slr0115, *rre31*) ORF was amplified by PCR using KOD Plus Neo polymerase (Toyobo, Osaka, Japan) and the specific primers 5′-GAATTATAACCATATGCCTCGAATACTGATC-3′ (forward) and 5′-ATCCAATGTGAGGTTAACCTACGTTGGACTACCGCC-3′ (reverse). The amplified PCR fragment was inserted into the *Nde*I-*Hpa*I sites of the pTKP2031V vector, using an In-Fusion HD cloning kit (Takara Bio, Shiga, Japan). The resultant plasmid was confirmed by sequencing and transformed into GT-I as described previously (Osanai et al., 2011).

### Construction of Plasmids for Protease-Knockout Mutants

To construct protease-knockout mutants, the coding regions of proteases were amplified by PCR with KOD Plus Neo and the following primer sets: *clpB1*(slr1641) 5′-GGGAATTCTGCGGGATCGCAAACTA-3′ (forward) and 5′-GCGCATGCGAGCGTTGAATGGCTTCG-3′ (reverse); *clpB2* (slr0156) 5′-GGGAATTCTCCGCGCGTTTAACCTTG-3′ (forward) and 5′-GCGCATGCCCCGCTCAGCTTTTTCT-3′ (reverse); *clpC*(sll0020) 5′-GGGAATTCGCTTCCTGCCCGATAAG-3′ (forward) and 5′-GCGCATGCCACGTCTTCCAACAGGC-3′ (reverse); *clpX*(sll0535) 5′-GGGAATTCGAAGGAACGGTGGCCAA-3′ (forward) and 5′-GCGCATGCCCGTCGTTGTCCAACCA-3′ (reverse); *degP*(slr1204) 5′-GGGAATTCGTGCTGGGGGGACATTT-3′ (forward) and 5′-GCGCATGCCAGTTTGCCCACTAGGG-3′ (reverse); *degQ*(sll1679) 5′-GGGAATTCCTTGGTTACGCCGCATC-3′ (forward) and 5′-GCGCATGCACTATGCGCTGTAGGCG-3′ (reverse); and *degS*(sll1427) 5′-GGGAATTCGTGGCCGTGCTTTTACT-3′ (forward) and 5′-GCGCATGCCTTCCACCCGTTCTTGA-3′ (reverse). PCR fragments were isolated with the Wizard SV Gel and PCR Clean-up System (Promega, Madison, WI, USA) and the fragments were digested with *Eco*RI and *Sph*I. Each of the resulting fragments was cloned into pUC119 (Clontech) digested with *Eco*RI and *Sph*I. The chloramphenicol-resistant cassette obtained by digesting pKRP10 (Reece and Phillips, 1995) with *Sma*I or *Pst*I was inserted into the *Hinc*II sites of *clpB1* and *clpC*, the SmaI sites of *clpB2* and *clpX*, and the *Pst*I sites of *degP*, *degQ*, and *degS*. The resultant vectors were transformed into GT. The chloramphenicol-resistant (20 μg/mL) cells were isolated and streaked on plates several times.

### Immunoblotting

Cells grown under light or dark conditions were collected by centrifugation (5,800 × *g* for 2 min), and the supernatant was removed by pipetting. The cells were frozen by liquid nitrogen. Cells were dissolved in PBS-T (137 mM NaCl, 2.7 mM KCl, 8.1 mM Na_2_HPO_4_, 1.5 mM KH_2_PO_4_, 0.1% Tween-20) and disruption by sonication and immunoblotting was performed as described previously (Osanai et al., 2014a). Antisera against SigE and GlgP(sll1356) were generated previously (Osanai et al., 2009, 2011).

### RNA Isolation and Quantitative Real-Time PCR

RNA isolation and cDNA synthesis were performed as described previously (Osanai et al., 2014b). The cDNAs were synthesized with the SuperScript III First-Strand Synthesis System (Life Technologies Japan, Tokyo, Japan) with 2 μg of total RNA. Quantitative real-time PCR was performed with the StepOnePlus Real-Time PCR System (Life Technologies) according to the manufacturer’s instructions, using the primers listed in Supplementary Table S2. The transcript level of *rnpB*, which encodes RNaseP subunit B, was used as an internal standard as previously described (Osanai et al., 2015).

### Glycogen Measurement

Glycogen levels were measured at the Biotechnology Center of Akita Prefectural University (Akita, Japan), as described previously (Osanai et al., 2014b).

### LC–MS/MS Analysis

Equal amounts of cells (10 mL of cell culture with OD_730_ = 1.0) were harvested by rapid filtration. LC–MS/MS analysis was performed using a 100-mL aliquot of the upper phase as previously described (Osanai et al., 2014b). All metabolite analyses were performed with the cells grown without external carbon sources except CO_2_.

### Amino Acid Analysis by GC–MS

Equal amounts of cells (50 mL of cell culture with OD_730_ = 1.0) were harvested by rapid filtration. Amino acids were quantified by GC-MS as previously described (Osanai et al., 2014a).

### Organic Acid Analysis by GC–MS

Equal amounts of cells (10 mL of cell culture with OD_730_ = 1.0) were harvested by rapid filtration using a previously described method (Osanai et al., 2014b). GC-MS was carried out using a GCMS-QP2010 Ultra equipped with a CP-Sil 8 CB-MS capillary column (30 m × 0.25 mm × 0.25 μm; Agilent, Palo Alto, CA, USA) as previously described (Osanai et al., 2014b).

## Results

### Slower Growth of *rpaA*-Overexpressing Strain

**FIGURE 1:**
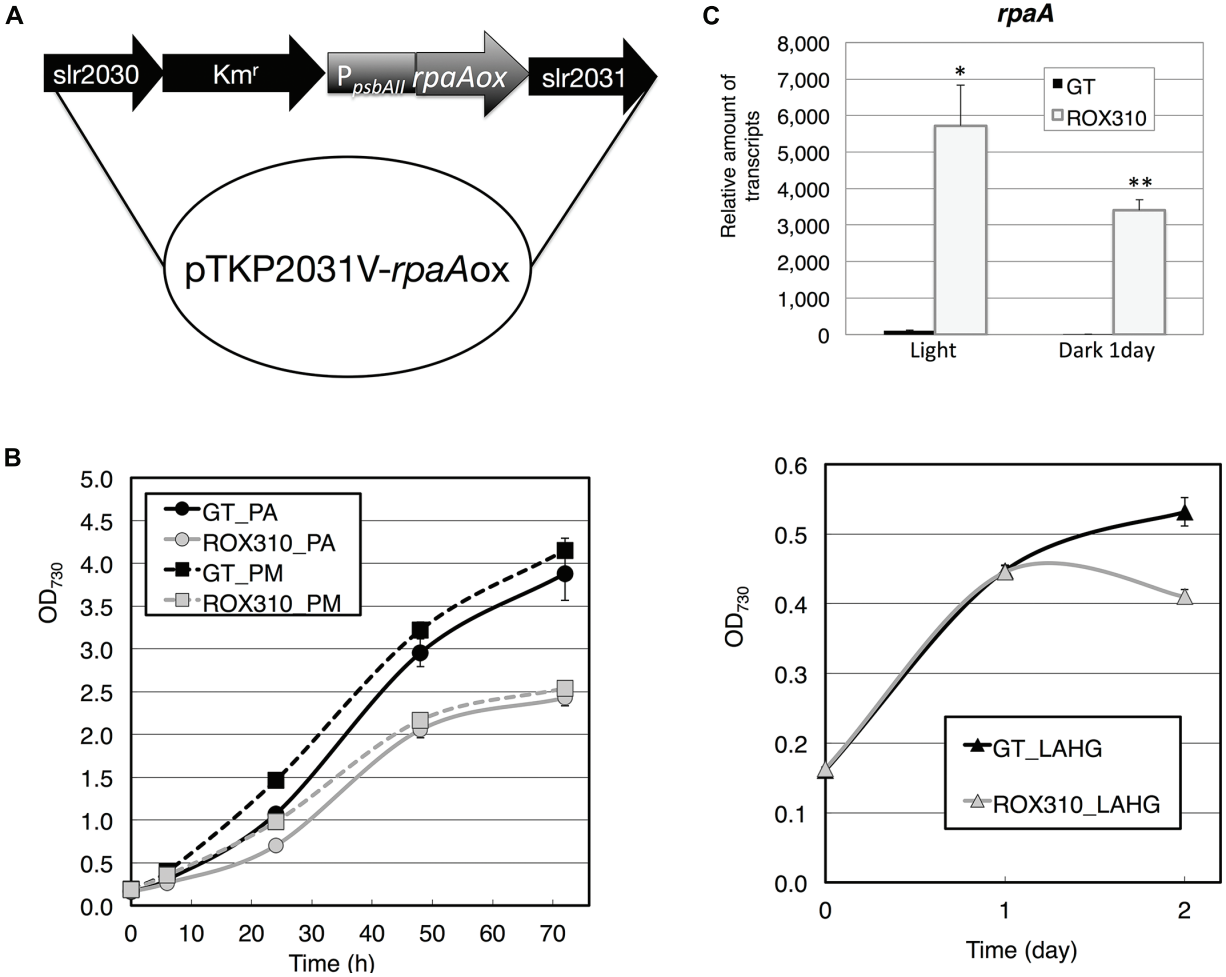
**(A)** Plasmid for *rpaA* overexpression in *Synechocystis* 6803. The kanamycin resistance cassette was located upstream of the *psbAII* promoter. **(B)** Levels of *rpaA* transcripts in GT and ROX310 cells. Quantitative real-time PCR was performed with total RNA from cells grown under light and dark conditions. **(C)** Growth of GT and ROX310 cells. Left: growth of cells under photoautotrophic (PA) and photomixotrophic (PM, grown with 1 mM glucose) conditions. Right: growth of cells under light-activated heterotrophic growth conditions (LAHG, grown with 1 mM glucose under continuous dark conditions except for 15 min light per day).

We generated a strain overexpressing *rpaA* by fusing the promoter of *psbAII* (encoding Photosystem II D1 protein; **Figure 1A**) and the strain was named ROX310. Quantitative real-time PCR confirmed that the expression levels of *rpaA* in ROX310 were higher than in the wild-type, glucose-tolerant (GT) strain under both light and dark conditions (**Figure 1B**). Under both photoautotrophic and photomixotrophic conditions, the *rpaA*-overexpressing strain grew more slowly than did GT (**Figure 1C**). ROX310 grew similarly to GT under light-activated heterotrophic growth (LAHG) conditions until 1 day, although it lost viability under prolonged LAHG conditions (**Figure 1C**).

### Altered Protein and Transcript Levels of SigE in ROX310

**FIGURE 2:**
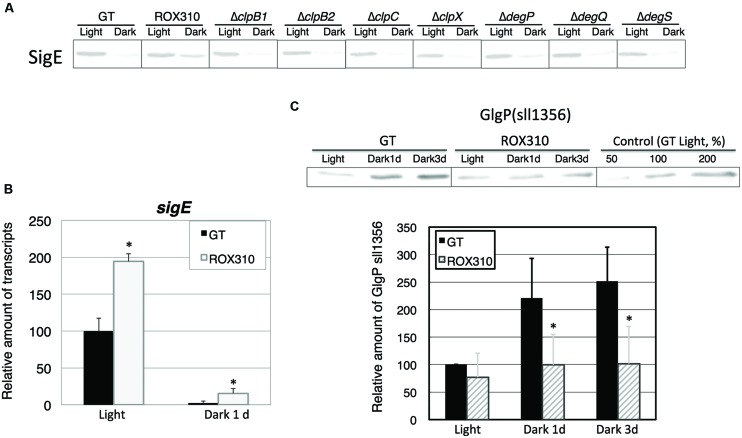
**(A)** Protein levels of SigE in GT, ROX310, and protease-knockout mutants. Immunoblotting was performed with 12 mg of total protein from cells grown under light and dark conditions (1 day). **(B)** Transcript levels of *sigE* in GT and ROX310. Quantitative real-time PCR was performed with total RNA from cells grown under light and dark conditions (1 day). Data represent mean ± SD from three independent experiments. (Student’s *t*-test; *P < 0.05). **(C)** Protein levels of GlgP(sll1356), one of two glycogen phosphorylases, under light and dark conditions. Immunoblotting was performed with 14 mg of total protein from cells grown under light and dark conditions (1 or 3 days). Protein levels were calibrated relative to that of the corresponding protein in GT (set at 100%). Data represent means ± SD from three independent experiments. Asterisks indicate statistically significant differences between GT and ROX310 (Student’s *t*-test; *P < 0.05).

Previously, the *hik8*-overexpressing strain showed aberrant protein degradation of SigE, RNA polymerase sigma factors activating sugar catabolism that is essential for dark/heterotrophic conditions, after the light-to-dark transition (Osanai et al., 2015). Immunoblotting demonstrated that *rpaA* overexpression reduced the degradation of SigE proteins under dark conditions (**Figure 2A**). To identify the proteases of SigE, we tested seven mutants lacking orthologous proteinases related to dark conditions in *Synechococcus* 7942 (Hosokawa et al., 2011), but these knockouts did not affect SigE protein levels (**Figure 2A**). The level of *sigE* transcripts was higher in ROX310 than in GT under both light and dark conditions, although the levels were similarly decreased in both strains by the light-to-dark transition (**Figure 2B**). We then quantified the levels of sugar catabolic enzymes by immunoblotting. The protein levels of GlgP(sll1356), one of two glycogen phosphorylases involved in glycogen degradation in *Synechocystis* 6803, were not induced by the *rpaA*-overexpressing strain under dark conditions (**Figure 2C**).

**FIGURE 3:**
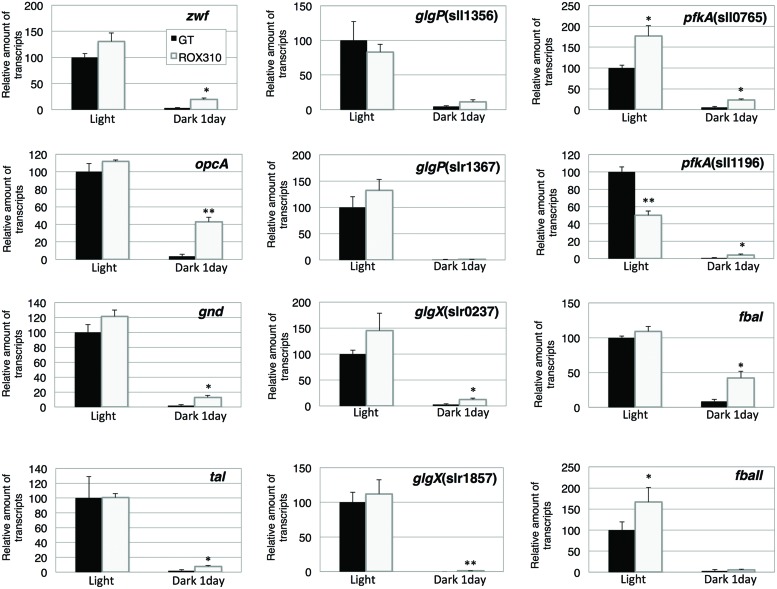
**Quantitative real-time PCR analysis of transcription in GT and ROX310**. Figure shows relative transcript levels of 12 genes involved in the OPP pathway (*zwf*, *opcA*, *gnd*, *tal*), glycogen catabolism [*glgP*(sll1356), *glgP*(slr1367), *glgX*(slr0237), *glgX*(slr1857)], and glycolysis [*pfkA*(sll0745), *pfkA*(sll1196), *fbaI*, *fbaII*]. Data represent mean ± SD from three independent experiments. Transcript levels were calibrated relative to that of the corresponding transcript in GT (set at 100%). Asterisks indicate statistically significant differences between GT and ROX310 (Student’s *t*-test; *P < 0.05, **P < 0.005).

We measured the transcript levels of genes related to sugar catabolism in ROX310 (**Figure 3**). The transcript levels of *pfkA*(sll0765) and *fbaII* were enhanced by *rpaA* overexpression under light conditions, while that of *pfkA*(sll1196) was repressed (**Figure 3**). The transcript levels of all 12 genes were decreased at 1 day after the light-to-dark transition in both GT and ROX310; however, there were higher transcript levels of these genes in ROX310 than in GT (**Figure 3**).

### Metabolome Analysis using the Cells Grown under Light and Dark Conditions

The glycogen levels were quantified under light and dark conditions. Glycogen rapidly disappeared in GT after the light-to-dark transition (**Table 1**). In ROX310, glycogen decreased after the light-to-dark transition, but the glycogen degradation was slower than GT (**Table 1**).

**Table 1:** Relative values of glycogen in GT and ROX310.

	Light	Dark 2 h	Dark 4 h	Dark 6 h
GT	100 ± 25.4	ND	ND	ND
ROX310	104 ± 32.7	21.9 ± 16.8	9.4 ± 6.4	ND

*Data represent mean ± SD from four independent experiments. Glycogen levels were calibrated relative to that in the GT strain under light conditions (set at 100%). ND indicates glycogen under detectable levels.*

LC–MS/MS analysis revealed that the levels of sugar phosphates (glucose-6-phosphate, ribose-5-phosphate, sedoheptulose-7-phosphate, fructose-6-phosphate, ribulose-5-phosphate, fructose-1,6-bisphosphate) and dihydroxyacetone phosphate were lower in ROX310 than in GT under both light and dark conditions (**Table 2**). Phosphoenolpyruvate levels were higher in ROX310 than in GT under light and dark conditions (**Table 2**). Fumarate and isocitrate levels were lower in ROX310 than in GT under light conditions (**Table 2**). Malate could not be detected in ROX310 under light conditions (**Table 2**). Organic acids in the TCA cycle were lesser in ROX310 than in GT under dark conditions (**Table 2**). NADP levels increased by *rpaA* overexpression, but other nucleotides did not (**Table 2**).

**Table 2:** Relative levels of metabolites in primary metabolism in GT and ROX310.

Metabolites	GT_Light	GT_Dark1day	ROX310_Light	ROX310_Dark1day
**Sugar phosphates and nucleotide sugars**				
Glucose-6P	100 ± 20.5	10.0 ± 1.3	33.3 ± 12.4*	0.8 ± 0.2**
Ribose-5P	100 ± 11.4	18.2 ± 4.4	115.9 ± 14.3	7.3 ± 1.9*
Sedoheptulose-7P	100 ± 12.7	5.4 ± 0.6	86.0 ± 13.1	2.5 ± 0.8*
Fructose-6P	100 ± 6.7	9.2 ± 1.0	54.3 ± 4.5**	1.2 ± 0.4**
Glucose-1P	100 ± 15.7	8.1 ± 3.7	41.1 ± 19.5*	4.9 ± 0.9
Glyceraldehyde-3P	100 ± 26.2	18.6 ± 7.3	99.7 ± 25.0	6.4 ± 3.3
Xylulose-5P	100 ± 19.7	11.0 ± 2.6	131.6 ± 22.8	6.4 ± 2.0
Ribulose-5P	100 ± 24.5	10.7 ± 2.0	143.3 ± 2.0	6.0 ± 1.2*
Fructose-1,6-bisP	100 ± 29.7	38.9 ± 7.8	82.1 ± 10.2	23.7 ± 3.7*
Ribulose-1,5-bisP	100 ± 20.6	34.4 ± 10.6	74.6 ± 13.1	18.3 ± 3.6
ADP-glucose	100 ± 43.8	2.7 ± 5.4	88.1 ± 22.8	0 ± 0
**Other metabolites in glycolysis and the OPP pathway**				
6-Phosphogluconate	100 ± 22.1	20.8 ± 6.5	83.8 ± 3.1	18.0 ± 2.9
DHAP	100 ± 13.6	23.9 ± 3.5	101.7 ± 18.8	8.1 ± 1.3
3- or 2-Phosphoglycerate	100 ± 5.4	25.3 ± 8.1	107.1 ± 2.1*	35.7 ± 8.6
Phosphoenolpyruvate	100 ± 7.5	18.6 ± 4.2	127.7 ± 7.8**	45.5 ± 13.9
Pyruvate^#^	100 ± 30.6	0 ± 0	113.9 ± 12.9	0 ± 0
Lactate^#^	100 ± 5.4	135.1 ± 26.4	149.9 ± 51.4	92.5 ± 13.8
**Metabolites in acetyl-CoA and the TCA cycle**				
Acetyl-CoA	100 ± 44.1	31.7 ± 6.1	71.1 ± 3.8	42.1 ± 5.3
Citrate^#^	100 ± 19.0	127.2 ± 13.1	96.4 ± 5.6	161.4 ± 24.2
Isocitrate^#^	100 ± 3.9	110.6 ± 8.2	63.3 ± 3.9**	64.2 ± 4.1**
Succinate^#^	100 ± 9.5	69.5 ± 4.2	83.3 ± 16.4	53.7 ± 9.6
Malate^#^	100 ± 89.6	14.9 ± 29.9	0 ± 0	32.5 ± 37.5
Fumarate^#^	100 ± 11.8	87.1 ± 32.2	19.7 ± 25.3*	68.8 ± 20.7
**Other cofactors**				
AMP	100 ± 20.4	92.7 ± 17.4	92.8 ± 7.2	107.4 ± 8.8
ADP	100 ± 10.3	107.7 ± 23.9	93.6 ± 6.5	122.9 ± 6.5
ATP	100 ± 12.5	117.6 ± 7.7	90.4 ± 5.9	112.8 ± 8.0
NAD	100 ± 3.8	137.7 ± 9.5	94.1 ± 5.5	124.4 ± 13.6
NADP	100 ± 8.7	161.5 ± 17.1	131.6 ± 7.6**	215.6 ± 9.6*
NADH	100 ± 108.7	55.8 ± 17.3	18.8 ± 22.5	34.9 ± 26.5*
NADPH	100 ± 31.3	52.8 ± 12.1	61.3 ± 11.3	35.2 ± 5.7

Data represent mean ± SD from four independent experiments. Metabolite levels were calibrated relative to that of the corresponding metabolite in GT under light conditions (set at 100%). Asterisks indicate statistically significant differences between GT and ROX310 (Student’s *t*-test; *P < 0.05, **P < 0.005). The data from the GT strain under light and dark conditions were cited from our previous study (Osanai et al., 2015). P, phosphate; DHAP, dihydroxyacetone phosphate. Metabolites were quantified by LC–MS/MS, except for those marked by #, which were quantified by GC–MS.

**FIGURE 4:**
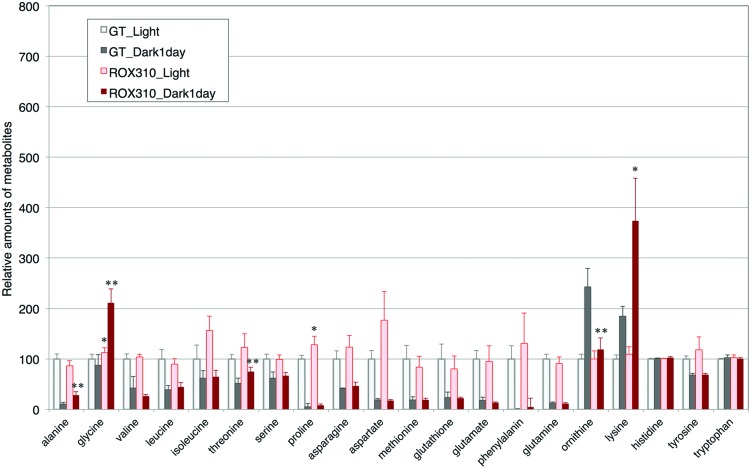
**Levels of 18 amino acids, ornithine, and glutathione in ROX310.** Data represent means ± SD from four independent experiments. Levels were calibrated relative to that of GT grown under light conditions (set at 100%). Asterisks indicate statistically significant differences between GT and ROX310 (Student’s *t*-test; *P < 0.05, **P < 0.005).

Amino acid analyses showed that the levels of glycine and proline were higher in ROX310 than in GT under light conditions (**Figure 4**). The levels of alanine, glycine, threonine, and lysine were higher in ROX310 than in GT under dark conditions (**Figure 4**). The level of ornithine was lower in ROX310 than in GT under dark conditions (**Figure 4**).

### Alteration in Transcript Levels of Genes Encoding Circadian Clocks

**FIGURE 5:**
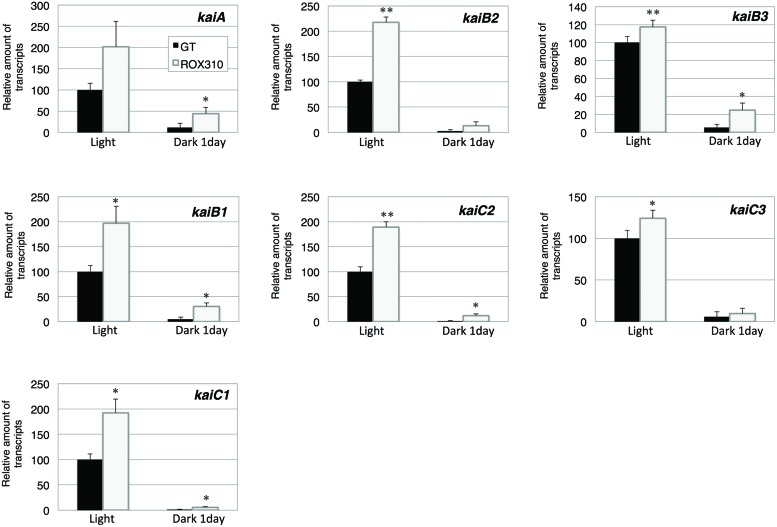
**Quantitative real-time PCR analysis of transcription in GT and ROX310**. Figure shows relative transcript levels of seven genes encoding KaiABC proteins. Data represent mean ± SD from three independent experiments. Transcript levels were calibrated relative to that of the corresponding transcript in GT (set at 100%). Asterisks indicate statistically significant differences between GT and ROX310 (Student’s *t*-test; *P < 0.05, **P < 0.005).

Finally, expression of *kaiABC* genes was quantified. The transcript levels of *kaiA, B1*, *C1*, *B2*, and *C2* were doubled by *rpaA* overexpression under light conditions (**Figure 5**). The transcript levels of *kaiB3* and *kaiC3* were marginally increased in ROX310 under light conditions (**Figure 5**). All the transcripts of *kaiABC* were decreased after 1 day of dark cultivation, and the levels remained higher in ROX310 than those in GT (**Figure 5**).

## Discussion

Here, we have performed genetic and metabolomic analyses using an *rpaA*-overexpressing strain and revealed that RpaA is involved in the regulation of primary metabolism in this unicellular cyanobacterium. The mechanism of circadian clocks in *Synechocystis* 6803, which could be different from *Synechococcus* 7942, has been less studied except Drs. Axmann’s and Hellingwerf’s groups (Wiegard et al., 2013; Beck et al., 2014; van Alphen and Hellingwerf, 2015), and we proceeded metabolome analyses with the circadian-related mutants of *Synechocystis* 6803. ChIP-Seq analysis in *Synechococcus* 7942 shows that RpaA binds the promoters of *kaiBC*, *sasA*, *rpaA*, and genes encoding sigma factors (*rpoD2, D5, D6*) and sugar catabolic enzymes (*glgP*, *malQ*, *zwf*, *opcA*, *gap1*, *fbaII*), activating their gene expression at night (Markson et al., 2013). These results are consistent with our analysis: the *rpaA* overexpression altered the gene expression of *sigE*, a sugar catabolic enzyme, and *kaiABC* (**Figures 2**, **3**, and **5**). SigE is a sigma factor activating glycogen catabolism, glycolysis, and the OPP pathway (Osanai et al., 2005, 2011), and its expression peaks before night (Kucho et al., 2005). Combining the results of previous and current genetic analyses, the signal transduction from the circadian clock to sugar metabolism consists of the cascade of the proteins KaiABC-Hik8-RpaA-SigE in *Synechocystis* sp. PCC 6803, although further promoter analysis is required. Since RpaA altered the expression of *kaiABC* genes (**Figure 5**), feedback or feedforward regulation from RpaA to the central circadian oscillator may exist to entrain the clock by metabolic information.

The *rpaA*-overexpressing strain of *Synechocystis* 6803 exhibited several phenotypes. Results from the *rpaA*-overexpressing strain of *Synechocystis* 6803 were similar to the *rpaA-*null mutant of *Synechococcus* 7942, which showed decreased glycogen catabolism (Diamond et al., 2015). The *rpaA* knockout mutant in *Synechocystis* 6803 exhibited high light sensitive phenotype (Majeed et al., 2012), which is consistent with our results that RpaA is important in light acclimation. A previous study demonstrated that introduction of RpaA(D53E; which mimics phosphorylated RpaA) restored the RpaA function, but that the introduction of RpaA(D53A; which mimics non-phosphorylated RpaA) could not (Markson et al., 2013). Therefore, the *rpaA* overexpression in our study may have increased non-phosphorylated RpaA in the *Synechocystis* 6803 cells, leading to phenotypes that were the mixture of gain-of-function and loss-of-function of RpaA. Introduction of a phospho-mimic RpaA into *Synechocystis* 6803 may be intriguing to distinguish these phenotypes.

Overexpression of *rpaA* led to phenotypes similar to those of the *hik8* overexpressor (Osanai et al., 2015); that is, growth defects under light-activated heterotrophic conditions (**Figure 1**), aberrant degradation of SigE after the light-to-dark transition (**Figure 2A**), accumulated transcripts during darkness (**Figure 3**), decreased levels of sugar phosphates (**Table 2**), and increased levels of several amino acids under dark conditions (**Figure 4**). These similarities may be due to the overexpression of *hik8* accelerated the phosphorylation of RpaA proteins. The glycogen metabolism mutant could not grow under dark/heterotrophic conditions (Osanai et al., 2005; Singh and Sherman, 2005; Tabei et al., 2007), or treatment with high salt and oxidative stress (Suzuki et al., 2010). Thus, the growth phenotypes of ROX310 under LAHG conditions may be caused by the changes in primary metabolism (**Figure 1C**). Genetic analyses have suggested that the mutants of Clp proteases alter circadian oscillation in *Synechococcus* 7942 (Holtman et al., 2005; Imai et al., 2013). Nevertheless the aberrant degradation of SigE proteins under dark conditions in the *rpaA*-overexpressing strain (**Figure 2A**), glycogen catabolism was slowed under dark conditions (**Table 1**). These results suggest that proteins other than SigE concertedly determine the degree of glycogen degradation in *Synechocystis* 6803. Several sugar catabolic regulators, including Hik31, Rre37, and AbrB are known in *Synechocystis* 6803 (Kahlon et al., 2006; Tabei et al., 2007; Yamauchi et al., 2011). For example, Rre37 preferentially activates the gene expression of glycogen catabolic and glycolytic enzymes such as *pfkA*(sll1196; Azuma et al., 2009), and thus, further study of the relationships among several sugar catabolic regulators is necessary to elucidate the regulatory mechanism of sugar catabolism in *Synechocystis* 6803.

Control of primary carbon metabolism by a circadian clock is an important theme in cyanobacteria (Diamond et al., 2015). Decrease in glycogen and sugar phosphates was observed during dark conditions (**Tables 1** and **2**), which indicates glycogen and sugar phosphates are positively correlated in this condition. On the other hand, organic acids in the TCA cycle kept higher levels under dark conditions (**Table 2**). Thus, organic acids in the TCA cycle were not correlated with the metabolites in glycolysis and the OPP pathway. The *rpaA*-overexpressing strain showed decreased levels of sugar phosphates under both light and dark conditions (**Table 2**), which is consistent with the fact that RpaA is important for the expression of genes related to glycogen catabolism in unicellular cyanobacteria (Diamond et al., 2015). Our immunoblotting showed that GlgP(sll1356) proteins were decreased with *rpaA* overexpression (**Figure 2C**), which may be one reason for the down-regulation of glycogen catabolism in this mutant. The metabolomic analysis also revealed that organic acids in the TCA cycle (fumarate, malate, and oxaloacetate) were lowered by *rpaA* overexpression (**Table 2**). The organic acids in the TCA cycle are an important pool of carbon sources in this cyanobacterium (Osanai et al., 2014a). Thus, the data also indicates that RpaA widely regulates primary metabolism related to the carbon sinks in this cyanobacterium. KaiC regulates the production of lysine, which has been shown to be lowered during light/dark cycles in *Synechococcus* 7942 (Diamond et al., 2015). The analysis showed lysine and glycine levels were up-regulated by *rpaA*-overexpression during the light-to-dark transition (**Figure 4**), demonstrating the involvement of RpaA in amino acid metabolism in response to light conditions. In summary, our metabolome analyses have revealed the RpaA-regulation in primary sugar and amino acid metabolism of *Synechocystis* 6803.

## Conflict of Interest Statement

The authors declare that the research was conducted in the absence of any commercial or financial relationships that could be construed as a potential conflict of interest.
