# Supplementary material for: Changes in primary metabolism under light and dark conditions in response to overproduction of a response regulator RpaA in the unicellular cyanobacterium Synechocystis sp. PCC 6803
Source: Front Microbiol. 2015 Aug 26;6:888. doi: 10.3389/fmicb.2015.00888 (PMC4549654; doi:10.3389/fmicb.2015.00888)
Supplement: Supplementary file 2 [file Table_2.DOCX]

**Table S2** List of primers

| Gene name | Forward primer | Reverse primer |
| --- | --- | --- |
| *rnpB* | 5'-AAAGGGTAAGGGTGCAAAGG-3' | 5'-AATTCCTCAAGCGGTTCCAC-3' |
| *zwf* | 5'-AGGCATTTCCCTGCGTTTT-3' | 5'-GCGGGTACGCAGTTCTGAA-3' |
| *opcA* | 5'-CAATGCGGCACCGTACTGT-3' | 5'-CAGCTTCCATGCGCATACAA-3' |
| *gnd* | 5'-CCCATCGCCGTGTTTAACC-3' | 5'-CGTTCGGCCATGAATTTCTC-3' |
| *tal* | 5'-CCGGCTGTGACCTGTTGAC-3' | 5'-CGGTGTTCCGCAGTTGATC-3' |
| *glgP*(sll1356) | 5'-CGGAACCCTACACCGATGAC-3' | 5'-ACCAAACTGCCGGCAATC-3' |
| *glgP*(slr1367) | 5'-ACCTTTGCCTACACCAATCACA-3' | 5'-ATCCACTGACCAGCGCTCTAG-3' |
| *glgX*(slr0237) | 5'-CTGGTGCGAGATACCAAAGGA-3' | 5'-TGGCGGTGGCAATTTCA-3' |
| *glgX*(slr1857) | 5'-TCCCTCCACTTCGATCAATTTT-3' | 5'-CCAAATCCGCCAGGGTAAA-3' |
| *pfkA*(sll0745) | 5'-GGTGGAACACGCTGACCTTAA-3' | 5'-GCGATTTTCCTGGTGACATTC-3' |
| *pfkA*(sll1196) | 5'-CGACACCGCCACCAACAT-3' | 5'-GCGGTGAAATGCAAACGAT-3' |
| *fbaI* | 5'-TTGGCCGCAATCCTGTTC-3' | 5'-CGTGGGCTCCCCATCAA-3' |
| *fbaII* | 5'-TAGCCCCGCCACTTGCTA-3' | 5'-CCATCATCACACTGGTGAAACC-3' |
| *sigE* | 5'-CCGCCTAGTGGTTTCCATTG-3' | 5'-CAGGTCGAGGAAGGGAACAC-3' |
| *kaiA* | 5'-GCCAAAACCACCGACGAA-3' | 5'-GCAGATGAATTTCAGCGTTGTG-3' |
| *kaiB1* | 5'-CACCCCCACCTTGGCTAAA-3' | 5'-GGTCGCCGATGATTTTCCT-3' |
| *kaiB2* | 5'-AAGTTTGGCAACTGCGTCTGT-3' | 5'-TTTATAAAAGCTGTGACGGATTTAGG-3' |
| *kaiB3* | 5'-GGCGATATGTCCAATACGGAAA-3' | 5'-GCAAACCTTCGGGCACAA-3' |
| *kaiC1* | 5'-GGAAGGTTGTCGCCAAAGAG-3' | 5'-GAGCCCTGGATTCCTCATAGG-3' |
| *kaiC2* | 5'-TTGGCGGGCAGGGTTAT-3' | 5'-CCAGCTCTCCCGGTTACTAAAA-3' |
| *kaiC3* | 5'-CCTCGCCGATGAAAAACG-3' | 5'-CGGTGCCACGGTATTTAAGG-3' |
| *rpaA* | 5'-TGCGCCGGGACGAA-3' | 5'-CAACGCCGTTAGCATCAAAA-3' |
